# Real-Time Electrical Bioimpedance Characterization of Neointimal Tissue for Stent Applications

**DOI:** 10.3390/s17081737

**Published:** 2017-07-28

**Authors:** David Rivas-Marchena, Alberto Olmo, José A. Miguel, Mar Martínez, Gloria Huertas, Alberto Yúfera

**Affiliations:** 1 Department of Electronics and Electromagnetism, Faculty of Physics, University of Seville, Av. Reina Mercedes sn, Seville 41012, Spain; david.rivas-marchena@ieee.org (D.R.-M.); gloria@imse-cnm.csic.es (G.H.); 2 Group of Microelectronics Engineering, Department of Electronics Technology, Systems Engineering and Automation, University of Cantabria, Santander 39005, Spain; jamd@teisa.unican.es (J.A.M.); martinez@teisa.unican.es (M.M.); 3 Seville Institute of Microelectronics, Microelectronics National Center, Consejo Superior de Investigaciones Científicas (IMSE-CNM-CSIC), Av. Americo Vespuccio, sn, Seville 41092, Spain; aolmo@dte.us.es; 4 Computer Engineering School (ETSII), University of Seville, Av. Reina Mercedes sn, Seville 41012, Spain

**Keywords:** bioimpedance, atherosclerosis, cardiology, oscillation-based test, stent

## Abstract

To follow up the restenosis in arteries stented during an angioplasty is an important current clinical problem. A new approach to monitor the growth of neointimal tissue inside the stent is proposed on the basis of electrical impedance spectroscopy (EIS) sensors and the oscillation-based test (OBT) circuit technique. A mathematical model was developed to analytically describe the histological composition of the neointima, employing its conductivity and permittivity data. The bioimpedance model was validated against a finite element analysis (FEA) using COMSOL Multiphysics software. A satisfactory correlation between the analytical model and FEA simulation was achieved in most cases, detecting some deviations introduced by the thin “double layer” that separates the neointima and the blood. It is hereby shown how to apply conformal transformations to obtain bioimpedance electrical models for stack-layered tissues over coplanar electrodes. Particularly, this can be applied to characterize the neointima in real-time. This technique is either suitable as a main mechanism for restenosis follow-up or it can be combined with proposed intelligent stents for blood pressure measurements to auto-calibrate the sensibility loss caused by the adherence of the tissue on the micro-electro-mechanical sensors (MEMSs).

## 1. Introduction

Ischemic heart disease is the leading cause of morbidity and mortality in the world [1,2]. The interior of the elastic and muscular arteries shrinks (stenosis), mainly by the growth of a lipid layer (atherosclerosis), causing angina chest pain and acute myocardial infarction. Among the existing treatments, the most popular employs a flexible metal mesh (stent) to mechanically support the opening of stenotic segments and ensure permeability [3,4]. However, despite improvements to the surgery techniques and the stent design [5,6,7], there is a high probability of medium-term restenosis [8]. 

In [9], the use of micro-electro-mechanical pressure sensors (MEMSs) to monitor restenosis with intelligent stents is proposed. The sensors are implemented as parallel-plate capacitors: one is a fixed plate, whereas the other bends with increasing pressure. The distance between the plates determines a capacitance that is read out with an electronic circuit [10]. However, the growth of the neointimal tissue on top of the flexible plate compromises its flexibility and degrades the sensor sensitivity.

Characterizing the neointima in real-time would ease the integration of an auto-calibration block in the readout circuit to compensate for the sensitivity loss of the MEMSs [11]. Different publications have recently emerged that use bioimpedance techniques to follow up the restenosis by studying the growth of new tissue in the artery (neointimal layer). Some solutions provide the catheter with a matrix of microelectrodes [12,13]. Other works place the electrodes directly on the stent [14]. It is also proposed to use the stent itself as a large electrode [15]. These alternatives still need catheterization to connect external measurement equipment, however.

**Figure 1:**
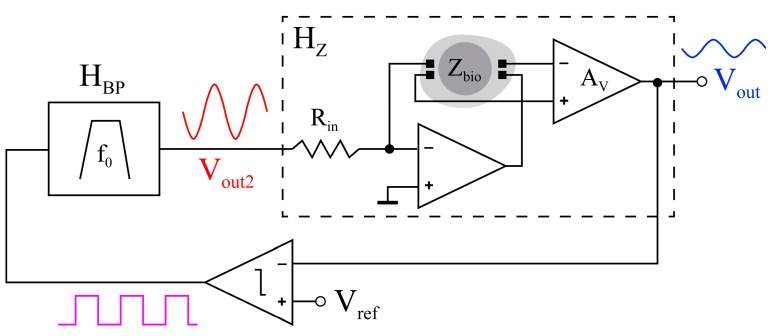
Bioimpedance self-stimulated monitoring system based on an oscillation-based test (OBT) [16]. H_BP_ is the Band-Pass filter, and Z_bio_ is the biological impedance.

A recently published method monitors cell-cultures in vivo based on their bioimpedance [16]. It consists of converting the cell-culture under test (CCUT) into a “biological oscillator”, whose characteristic parameters (i.e., frequency, amplitude, phase, etc.) are related with the cell-culture evolution and can be easily determined. This approach, known as an oscillation-based test (OBT), is also valid for monitoring the neointimal tissue growth. A system topology, such as in Figure 1, amplifies the bioimpedance and limits the outcome signal amplitude with a non-linear comparator. Closing the loop, a filter imposes a quasi-sinusoidal signal to stimulate the tissue. The input resistor keeps the current injected to the blood vessel within safe margins, typically below 20 μA, for cell protection [16]. At the node *V_out2_*, the oscillation is analyzed, and all the relevant parameters are extracted.

A solid understanding of the biological model is essential to link the experimental results with the neointima variations. This paper is primarily focused on the development of such an analytical model for the neointima, along with providing early proof-of-concept simulations for the applicability of OBTs to the real-time characterization of neointimal tissue variations. In the Materials and Methods section, a mathematical model of the neointima bioimpedance is presented from the different tissues comprising it. The model is compared to finite element analysis (FEA) simulations, and the validity of the model is shown in Section 3, along with a comparison between the analytical and simulated data. The results are further discussed in the final section, for generalizing the model and proposing improvements to it.

## 2. Materials and Methods 

### 2.1. Analytical Model

**Figure 2:**
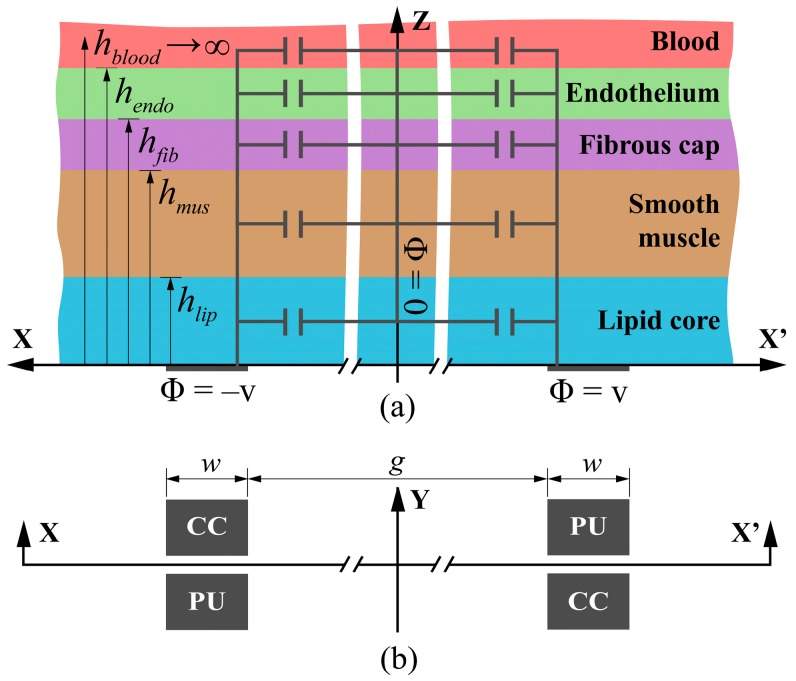
(**a**) Stack of neointimal tissue layers and parallel partial capacitances. (**b**) Layout of current-carrying (CC) and pick-up (PU) electrodes.

The neointima bioimpedance depends on the thickness of the tissue layers that comprise it—the lipid core, smooth muscle, fiber cap and endothelium. These are assumed to be stacked in parallel and immersed in blood, as in Figure 2a. Selecting a four-electrode setup [17], the bioimpedance value of the neointima layer is incorporated directly into the proposed system performance, without the need to consider the electrode impedance contributions in a first analysis of the system, as happens when using two-electrode setups [16]. The performance limitations imposed by the influence of electrodes in tetrapolar topologies should be considered for a correct circuit design. These electrodes are placed, in our design, around the MEMS, as shown in Figure 2b, to maximize the transference impedance.

#### 2.1.1. Neointima–Blood Interface (“Double Layer”)

Blood is an electrolyte—it has free charge carriers—but the cellular structure immobilizes charge in the neointima. The difference between the media produces a charge zone in which the blood ions that are closer to the interface are fixed by the outermost tissue layer [18]. This charge zone extends through the electrolyte, creating a capacitor that is included in the analytical model. The use of a triple-layer model [19] to describe the charge zone results in a relative permittivity of *ε_r_* = 17.8 and a Debye length of 0.64 nm (4 to 5 orders of magnitude smaller than the usual tissue thicknesses). Further influence of the intra-arterial blood pressure (depending on volume and flow) on electrical impedance is studied in [20], although an analytical model explaining this relation was not presented therein and should be object of future work.

#### 2.1.2. Dielectric Interface

From the set of Maxwell equations, it is derived that the electric field angle (*α*) in a dielectric interface changes by

(1) $$\frac{\tan\alpha_{2}}{\tan\alpha_{1}}=\frac{E_{2,t}/E_{2,n}}{E_{1,t}/E_{1,n}}=\frac{E_{1,n}}{E_{2,n}}=\frac{D_{1,n}/\varepsilon_{1}}{D_{2,n}/\varepsilon_{2}}=\frac{\varepsilon_{2}}{\varepsilon_{1}}$$

where **E_i,t_** and **E_i,n_** are the tangential and normal components of the electric field and **D_i,n_** are the normal components of the electric displacement for each dielectric. For interfaces at which the media permittivity varies largely, two different boundary conditions may apply:

- ***Dirichlet Boundary Condition (DBC)**, for ε*_2_ ≫ *ε*_1_—constant electric potential at the interface (Φ = 0).
- ***Neumann Boundary Condition (NBC)**, for ε***_2_** ≪ *ε*_1_—no potential gradient normal to the interface (∂Φ⁄∂**n** = 0).

#### 2.1.3. Partial Capacitance Method

Ghione and Goano [21] presented that the total equivalent capacitance between coplanar metal tracks in printed circuit boards (PCB) can be derived from the partial contribution of each layer of material deposited in parallel with them.

The electrodes of the intelligent stent are so close, in comparison with the stent diameter, that they can also be assumed to be coplanar; the neointima stratified in parallel layers of tissue are stacked on top of them (Figure 2a).

If the permittivity decreases with the distance to the electrode plane, all the dielectric interfaces can be modeled with NBCs. Then, the partial capacitances are effectively connected in parallel (parallel partial capacitance—PPC), so that the equivalent capacitance is

(2) $$C_{eq}=\sum_{i=1}^{i=n-1}\left[\varepsilon_{r,i}-\varepsilon_{r,i+1}\right]\;C_{PPC}(h_{i})+\varepsilon_{r,n}\;C(\infty )$$

where *n* is the number of layers, *h_i_* is the height of each layer measured from the electrode plane, and *C_PPC_(h_i_)* is the void capacitance of each layer limited by a NBC.

For the opposite, if the permittivity increases, the partial capacitances are assumed to be in series (series partial capacitance—SPC) with DBCs at the interfaces. In this case, the equivalent capacitance is

(3) $$\frac{1}{C_{eq}}=\sum_{i=1}^{i=n-1}\left[\frac{1}{\varepsilon_{r,i}}-\frac{1}{\varepsilon_{r,i+1}}\right]\;\frac{1}{C_{SPC}(h_{i})}+\frac{1}{\varepsilon_{r,n}}\frac{1}{\;C(\infty )}$$

Because the plates of these capacitors—the electrodes—are coplanar, there is no simple formula to compute capacitances. To cope with this, Igreja and Dias [22] apply conformal mapping techniques to transform the disposition of electrodes into a parallel-plate configuration, whose capacitance is

(4) $$C_{partial}=\varepsilon_{r}\;\varepsilon_{0}\;L\;\frac{K(k)}{K(k')}$$

and which depends on the tissue relative permittivity *ε_r_*, the electrode depth *L* and the equivalent width, derived from the mathematical transformation and described as a ratio of complete elliptic integrals of the first kind (*K*) with modulus *k* (see Table 1) and its complementary *k'* = √(1 − *k*^2^).

#### 2.1.4. Iterative Complex Method

The proposed PC method is only valid for stacks of material whose permittivities vary monotonically. Blume [23] shows an iterative method compatible with any kind of stack that groups layers in pairs and computes equivalent permittivities:

(5) $$\text{SPC}:\;\varepsilon_{eq,i}=\frac{\varepsilon_{eq,i-1}\;\varepsilon_{i+1}\;\kappa (h_{i})}{\kappa (h_{i+1})\left(\varepsilon_{i+1}-\varepsilon_{eq,i-1}\right)+\varepsilon_{eq,i-1}\;\kappa (h_{i})}$$

(6) $$\text{SPC}:\;\varepsilon_{eq,i}=\left(\varepsilon_{eq,i-1}-\varepsilon_{i+1}\right)\;\frac{\kappa (h_{i})}{\kappa (h_{i+1})}+\varepsilon_{i+1}$$

where *κ = K(k)*/*K(k’)* is used to simplify the expressions.

By replacing the permittivity with the complex permittivity (ε^*^ = *ε* − j*σ*/*ω*), the iterative method can calculate the equivalent impedance of any stack of parallel tissue layers as

(7) $$Z=j\;\omega /\;\left[L\;\kappa (\infty )\;\varepsilon_{\mathrm{eq},n-1}^{*}\right]$$

**Figure 3:**
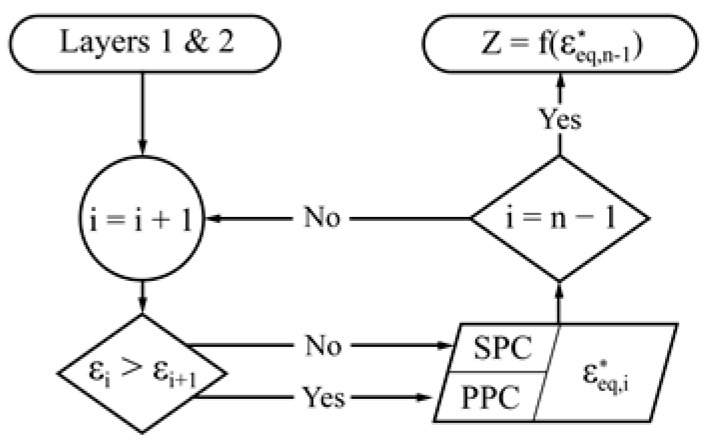
Iterative method to derive the analytical bioimpedance model.

A diagram of the iterative method is shown in Figure 3. The number of iterations that the method needs to complete is equal to the number of stacked tissues minus 1. This is implemented in MATLAB [24] and uses mainly the permittivity and conductivity data compiled by Gabriel and Gabriel [25].

**Figure 4:**
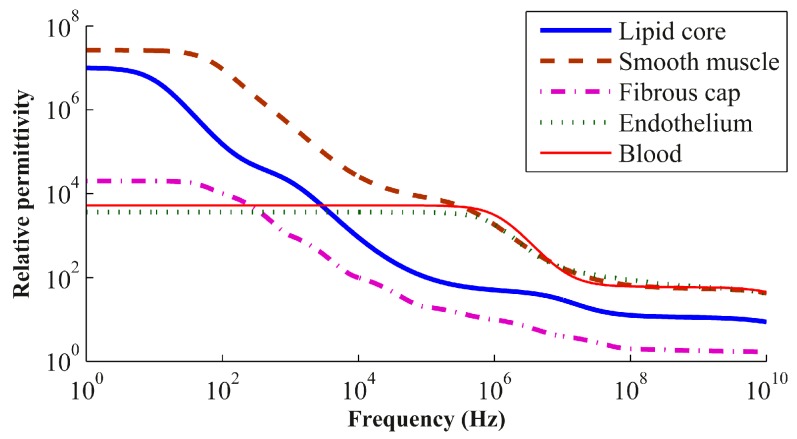
Relative permittivity versus frequency for the blood and neointimal tissues.

The comparison between permittivities (Figure 4) determines the frequency range for which the iterative method should apply SPC or PPC. Therefore, the method is predicted to be precise where *ε*_2_ ≫ *ε*_1_ or *ε*_2_ ≪ *ε*_1_ (clearly defined boundary conditions), as well as where *ε*_2_ ≈ *ε*_1_ (nearly no dielectric interface) [13]. 

The increment in total thickness after adding the “double layer” is quite small. This leads to similar elliptic integrals whose difference is comparable to the methodical error introduced by their analytical calculation. Hence, the results need an experimental adjustment to improve the accuracy.

### 2.2. Finite Element Analysis Simulations

The analytical bioimpedance model described previously was verified against FEA with COMSOL Multiphysics software [26]. Several sample configurations aimed to emulate the growth of neointimal tissue. In Table 2 is a list of tissue thicknesses for the different configurations.

The permittivity of the endothelium was much larger than that derived for the double layer (*ε_r_* = 17.8). Hence, this charge-zone effect could be modeled in the FEA simulation as an impedance contact condition between the endothelium and the blood [27]. 

**Figure 5:**
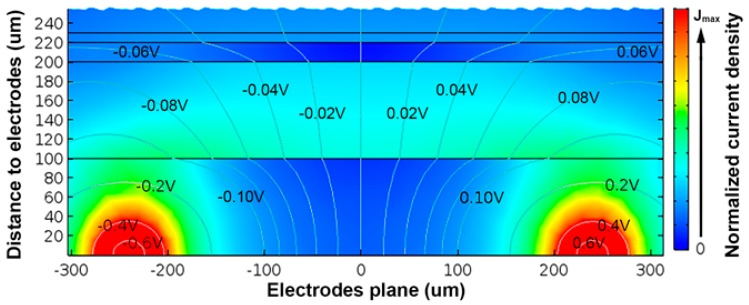
Two-dimensional finite element analysis (FEA) simulation model for the configuration #5 in Table 2. Electrodes were placed on the bottom left and right corners of the image (modeled without thicknesses). The color plane represents the normalized current density, whereas the grey lines are equipotentials (V). Higher current densities near the two electrodes can be observed.

The simplest model of the system is a 2D cross-section, as shown in Figure 5. This replicates the geometry of the mathematical transformations. The same assumptions made during the theoretical analysis are repeated here (i.e., no border effects, same electric fields and current in all the cross-sections, etc.). Hence, the validity of the bioimpedance can be easily proved against a simulation with the same limitations.

Only two electrodes were modeled, with no thickness, to dismiss the perturbation that their interface with the neointima would introduce. We note that this perturbation was minimized with the four-electrode configuration chosen. These electrodes were used for both injecting AC voltage to stimulate the tissues and to pick up the bioimpedance—derived by integrating along the current lines between electrodes.

The mesh was automatically created by the software, following a sequence controlled by the physics with a fine element size. This resulted in around 8000 triangles for the 2D model and 40,000 tetrahedrons in the 3D model. Their size shrunk around the boundaries. 

## 3. Results

### 3.1. Analytical Model and 2D Finite Element Analysis Simulation Results

**Figure 6:**
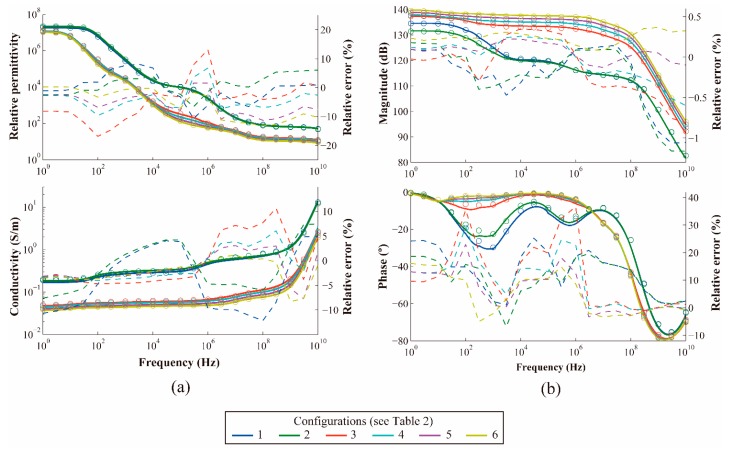
Comparison of the analytical model (circles) and 2D FEA simulation (solid). (**a**) Relative permittivity and conductivity. (**b**) Bioimpedance. In (**a**) and (**b**), dashed lines represent the relative error.

The histological configurations of Table 2 were sequentially fed into the iterative analytical method to derive the equivalent permittivity and conductivity of the neointima shown in Figure 6a. It was observed that the relative error for the six configurations was small and remained inside the expected accuracy, given the error introduced by mathematical transformations and the ideal boundary conditions.

The final equivalent bioimpedance was derived by also computing the effect of the blood. At this stage, an experimental adjustment was required to overcome the limitations of handling thin layers such as the double layer, as mentioned in the Analytical Model section.

The comparison between the analytical model and the 2D FEA simulation results is depicted in Figure 6b. The accuracy of the impedances is consistent with the relative errors computed for the equivalent permittivities and conductivities, related by Equation (7). We can observe a larger relative difference in phase than in magnitude, but it is a manageable difference for practical applications, such as the analysis of cell growth, by tuning the frequency of oscillations appropriately [16].

### 3.2. Generalization of the Bioimpedance Model

So far, the analytical model requires executing the iterative method for each histological configuration. However, based on several samples, a general expression for the bioimpedance electrical model in terms of its frequency dependence can be derived. It is assumed to have a gain, three poles and three zeroes, such that

(8) $$Z_{eq}(s=j\omega )=A\frac{\left(s+z_{1})(s+z_{2})(s+z_{3}\right)}{\left(s+p_{1})(s+p_{2})(s+p_{3}\right)}$$

By running least-squares fitting on the data samples, the third zero is obtained to be fixed at 20 GHz, whereas the gain, the poles and the remaining zeroes depend linearly on the thickness of each layer of neointimal tissue:

(9) $$x=\alpha_{1}\;d_{lip}+\alpha_{2}\;d_{mus}+\alpha_{3}\;d_{fib}+\alpha_{4}$$

where *x* is the parameter (gain, pole or zero), α*_i_* are the linear coefficients and *d_j_* are the thicknesses of the lipid, muscle and fiber layers. Different linear coefficients are computed for histological configurations without neo-atherosclerosis (i.e., no lipid core or fibrous cap), shown in Table 3 and, with it, Table 4.

### 3.3. Oscillation-Based Test Auto-Calibration Circuit

The set of histological configurations can be enlarged to that contained in Table 5. This recreates the usual case in which the patient firstly develops a neointima without atherosclerosis. Two subsets of configurations with neo-atherosclerosis show the growth of a fibrous cap over thin and thick muscle layers.

**Figure 7:**
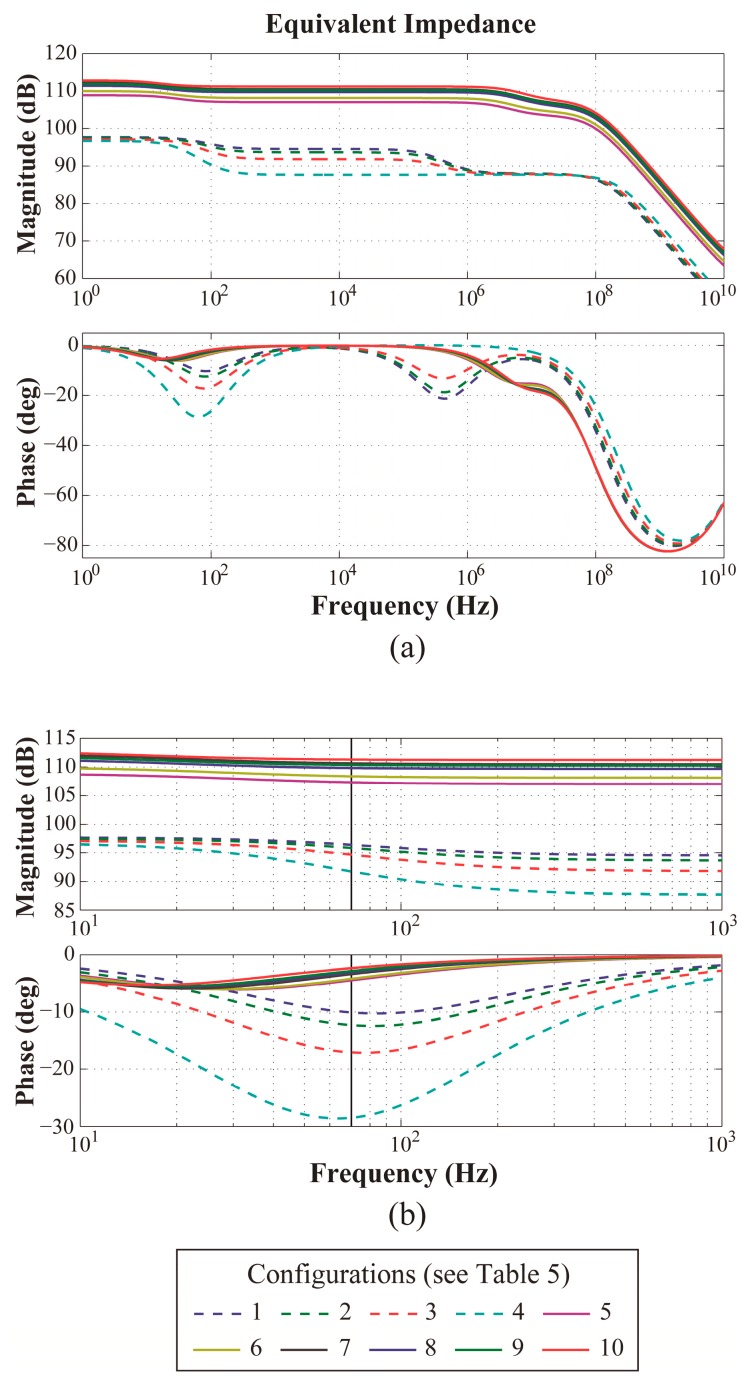
Bioimpedance of neointima for the histological configurations in Table 5: (**a**) large Bode plot, and (**b**) detailed plot at around 70 Hz.

**Figure 8:**
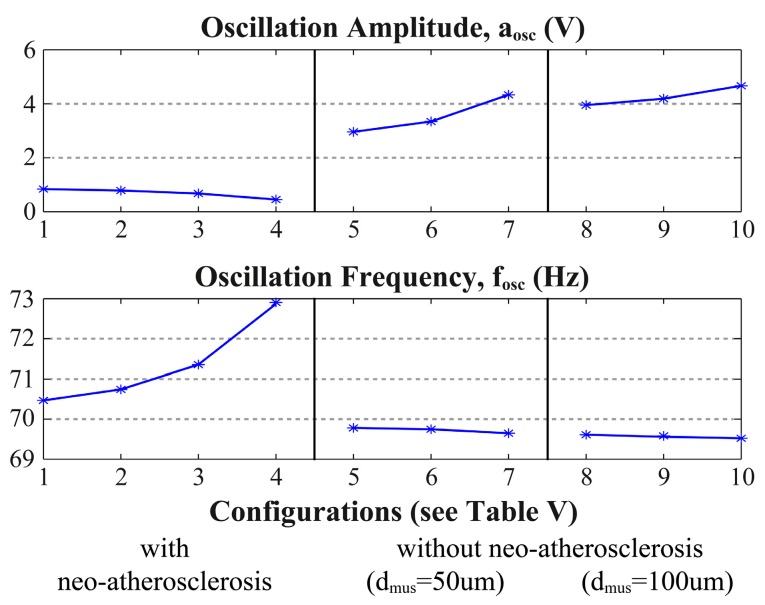
OBT auto-calibration circuit amplitude and frequency for the histological configurations in Table 5, measured at node *V_out2_* of Figure 1.

An interesting frequency to implement the OBT at is around 70 Hz, as there are large phase variations in this area, as depicted in Figure 7. The circuit loop depicted in Figure 1 was implemented in MATLAB for this frequency, as in [8]. The changes in neointimal histology caused the variations in oscillation amplitude and frequency shown in Figure 8.

These results prove the detection of neo-atherosclerosis by comparing the oscillation frequency with its nominal value. Additionally, changes in the oscillation amplitude help to distinguish the growth of different neointimal tissue layers. To simplify the measurements, the range of the oscillation frequency variation can be enlarged by changing the quality factor of the band-pass filter.

## 4. Discussion

An analytical method has been presented to obtain the bioimpedance of a stack of parallel tissues over coplanar electrodes, depending on the thickness of each layer and their electromagnetic properties. It consists of an iterative approach based on conformal spatial transformations. The correlation obtained between the analytical model and 2D FEA simulations proves to be enough for predicting the evolution of the bioimpedance with a reasonable accuracy and minimal computational cost. Based upon these results, Section 3.2 and Section 3.3 delved into the application of the analytical model to monitor the restenosis in vivo.

The analytical bioimpedance model gives a good prediction for the incremental changes in the neointima layer because the output circuit response obtained (frequency and amplitude) is monotonically dependent on neointima impedance changes. A calibration technique, for which some initial OBT reference values are known, allows for the identification of variations in the neointimal histology by observing increments in the amplitude and/or frequency of the oscillation, which could be used in the real-time monitoring of neointimal tissue in stent applications.

Further work is required to improve the overall system accuracy, including for border effects, anisotropy and other 3D non-idealities. Finally, a workaround to incorporate very thin layers, such as the double layer, or to incorporate effects of blood volume and flow to the analytical model is needed.

## Figures and Tables

**Table 1:** Conformal mapping parameters [21].

Series Partial Capacitance (SPC)	Parallel Partial Capacitance (PPC)
$k_{SPC}=\sqrt{\frac{t_{4}-t_{3}}{t_{4}-1}}$	$k_{PPC}=\frac{1}{t_{3}}\sqrt{\frac{t_{4}^{2}-t_{3}^{2}}{t_{4}^{2}-1}}$
$t_{3}=\cosh\left(\frac{\pi \;(1-\eta )}{4\;r}\right)$	$t_{3}=\cosh\left(\frac{\pi \;(1-\eta )}{8\;r}\right)$
$t_{4}=\cosh\left(\frac{\pi \;(1+\eta )}{4\;r}\right)$	$t_{4}=\cosh\left(\frac{\pi \;(1+\eta )}{8\;r}\right)$
η=w/(w+g)	
r=2 h/(w+g)	

In the equations, *w* and *g* are the width of the electrodes and the gap between them, respectively, and *h* is the height of a given tissue layer.

**Table 2:** Configurations of thicknesses for neointimal tissue layers.

Config.	Lipid Core	Muscle	Fibrous Cap	Endothelium
**1**	0 µm	25 µm	0 µm	10 µm
**2**	0 µm	50 µm	0 µm	10 µm
**3**	25 µm	50 µm	5 µm	10 µm
**4**	50 µm	100 µm	10 µm	10 µm
**5**	100 µm	100 µm	20 µm	10 µm
**6**	200 µm	100 µm	40 µm	10 µm

**Table 3:** Coefficients for Equation (9) in configurations without neo-atherosclerosis.

Param.	Lipid Core (*α*_1_)	Muscle (*α*_2_)	Fibrous Cap (*α*_3_)	Independent (*α*_4_)
***A***	—	4.60 × 10^−1^	—	1.72 × 10^2^
***p_1_***	—	−1.17 × 10	—	4.52 × 10^2^
***p_2_***	—	2.45 × 10^3^	—	1.82 × 10^6^
***p_3_***	—	2.56 × 10^6^	—	8.59 × 10^8^
***z_1_***	—	2.30 × 10^–1^	—	6.28 × 10^2^
***z_2_***	—	−9.72 × 10^3^	—	4.24 × 10^6^

**Table 4:** Coefficients for Equation (9) in configurations with neo-atherosclerosis.

Param.	Lipid Core (*α*_1_)	Muscle (*α*_2_)	Fibrous Cap (*α*_3_)	Independent (*α*_4_)
***A***	1.17	4.41	2.30 × 10^−1^	4.14 × 10^2^
***p_1_***	−1.70 × 10^−1^	−9.50 × 10^−1^	−3.00 × 10^−2^	2.24 × 10^2^
***p_2_***	3.02 × 10^4^	1.80 × 10^5^	6.03 × 10^3^	1.36 × 10^7^
***p_3_***	4.40 × 10^4^	2.99 × 10^5^	8.80 × 10^3^	5.46 × 10^8^
***z_1_***	−2.10 × 10^−1^	−1.23	−4.00 × 10^−2^	2.80 × 10^2^
***z_2_***	−4.79 × 10^4^	2.86 × 10^5^	9.57 × 10^3^	1.97 × 10^7^

**Table 5:** Extended configurations of thicknesses of neointimal tissue layers.

Config.	Lipid Core	Muscle	Fibrous Cap	Endothelium
**1**	0 µm	25 µm	0 µm	10 µm
**2**	0 µm	50 µm	0 µm	10 µm
**3**	0 µm	100 µm	0 µm	10 µm
**4**	0 µm	200 µm	0 µm	10 µm
**5**	25 µm	50 µm	5 µm	10 µm
**6**	100 µm	50 µm	20 µm	10 µm
**7**	300 µm	50 µm	50 µm	10 µm
**8**	50 µm	100 µm	10 µm	10 µm
**9**	100 µm	100 µm	20 µm	10 µm
**10**	200 µm	100 µm	40 µm	10 µm
